# Differential Dynamic and Structural Behavior of Lipid-Cholesterol Domains in Model Membranes

**DOI:** 10.1371/journal.pone.0040254

**Published:** 2012-06-29

**Authors:** Luis F. Aguilar, José A. Pino, Marco A. Soto-Arriaza, Francisco J. Cuevas, Susana Sánchez, Carlos P. Sotomayor

**Affiliations:** 1 Instituto de Química, Pontificia Universidad Católica de Valparaíso, Valparaíso, Chile; 2 Facultad de Química, Pontificia Universidad Católica de Chile, Santiago, Chile; 3 Departamento de Ciencias Básicas, Universidad Santo Tomás, Viña del Mar, Chile; 4 Microscopy and Dynamic Imaging Unit, National Center for Cardiovascular Research (CNIC), Madrid, Spain; 5 Laboratory for Fluorescence Dynamics, Department of Biomedical Engineering, University of California Irvine, Irvine, California, United States of America; Nagoya University, Japan

## Abstract

Changes in the cholesterol (Chol) content of biological membranes are known to alter the physicochemical properties of the lipid lamella and consequently the function of membrane-associated enzymes. To characterize these changes, we used steady-state and time resolved fluorescence spectroscopy and two photon-excitation microscopy techniques. The membrane systems were chosen according to the techniques that were used: large unilamellar vesicles (LUVs) for cuvette and giant unilamellar vesicles (GUVs) for microscopy measurements; they were prepared from dipalmitoyl phosphatidylcholine (DPPC) and dioctadecyl phosphatidylcholine (DOPC) in mixtures that are well known to form lipid domains. Two fluorescent probes, which insert into different regions of the bilayer, were selected: 1,6-diphenyl-1,3,5-hexatriene (DPH) was located at the deep hydrophobic core of the acyl chain regions and 2-dimethylamino-6-lauroylnaphthalene (Laurdan) at the hydrophilic-hydrophobic membrane interface. Our spectroscopy results show that (i) the changes induced by cholesterol in the deep hydrophobic phospholipid acyl chain domain are different from the ones observed in the superficial region of the hydrophilic-hydrophobic interface, and these changes depend on the state of the lamella and (ii) the incorporation of cholesterol into the lamella induces an increase in the orientation dynamics in the deep region of the phospholipid acyl chains with a corresponding decrease in the orientation at the region close to the polar lipid headgroups. The microscopy data from DOPC/DPPC/Chol GUVs using Laurdan generalized polarization (Laurdan GP) suggest that a high cholesterol content in the bilayer weakens the stability of the water hydrogen bond network and hence the stability of the *liquid-ordered phase* (*Lo*).

## Introduction

In addition to acting as a barrier for selective permeation, biological membranes act as a dynamic matrix that influences the integral proteins immersed in the bilayer. This matrix-protein interaction enables the membrane to carry out its distinctive physiological activity. The proposed influence of the structural and dynamic properties of the lamella on the activity of different proteins is widely accepted [1]; for instance, conformational changes of lipid acyl chains can determine the spatial distributions of the lipid components and strongly affect membrane functions [2]. Despite the existing evidence, the molecular details underlying the process remain poorly understood.

Cholesterol is ubiquitous in animal cell membranes and is one of the major modifiers of membrane structure and dynamics [3]. Changes in cholesterol content are known to alter the properties of the lipid lamella, which influences the functions of membrane-associated enzymes [4]. The effect of cholesterol on some of these properties (lamella hydration, orientational dynamics and the order of the phospholipids molecules) is the topic of this work. Considerable evidence suggests that the amount of water penetration into lipid bilayers varies from within the glycerol backbone to deeper within the fatty acyl chain packing defects; this differential penetration generates a hydration gradient and thus a dielectric constant gradient [5]. Thus, the ability of cholesterol to reduce the amount of water associated with the lipid bilayer is widely accepted [6]. The orientational dynamics of the phospholipids can be described by two types of motions: fast stochastic torsional librations and slow rotational motions [7]. Cholesterol suppresses the rotational motion and enhances the fast stochastic librations [8]. Librational motions may be relevant to chain dynamics in the high-density packing found in membrane raft domains [7]. In addition to the two well-known *gel* (*So*) and *liquid-crystalline* (*Ld*) phases, cholesterol may promote the formation of a third phase that coexists with the other two [9]–[11]; this phase is known as the “*liquid-ordered” phase* (*Lo*). This intermediate fluid phase exhibits translational degrees of freedom that are similar to the ones found in a conventional fluid lamellar state, whereas the conformational degrees of freedom of the lipid hydrocarbon chains resemble those of the gel state. The existence of this Lo phase provides the basis for the hypothesis that segregated fluid domains could coexist in biological membranes, i.e., the raft hypothesis [12]. Recently, atomic-scale molecular dynamics simulations explained the ability of cholesterol to increase order as the result of a collective and cooperative action of cholesterol molecules; this explanation emphasizes the importance of cholesterol’s unique molecular structure in promoting the formation of transient and local three-fold symmetric cholesterol-cholesterol structures that facilitate the formation of the liquid-ordered phase [13].

One proposed role for cholesterol in animal plasma membranes is to reduce the intrinsic tendency of glycerol and sphingolipids to form separate Lα and Lβ phases [14]. Indeed, a recent report showed that severe cholesterol depletion in the plasma membranes of living cells induces large-scale domain separation, presumably consisting of glycerophospholipid-enriched Lα and sphingolipid-enriched Lβ phases [15]. It is noteworthy that in the raft hypothesis, cholesterol facilitates a fluid–fluid lateral phase separation of the glycerophospholipid- and sphingolipid-enriched phases while simultaneously inhibiting the formation of sphingolipid-enriched Lβ phases.

Interactions between cholesterol and phospholipids are stabilized by hydrogen bonds (between the hydroxyl group of cholesterol and the phosphate and carbonyl groups of lipids) and by interactions between the choline group and the hydroxyl oxygen of cholesterol [16], [17]. In contrast, Bhattacharya and Haldar reported that the effect of cholesterol on some structural properties of the bilayer is independent of the ability of the lipids to form direct hydrogen bonds with the polar group of cholesterol [18]. However, several hydrogen bond interactions that are mediated by water have been proposed between phospholipids and cholesterol; the interactions form an extensive network at the lipid headgroups and play an important role in bilayer stability [19].

In the present study, we investigated the effect of cholesterol content on the structural and dynamic properties of bilayers formed by DOPC, DPPC and a 1∶1 DOPC/DPPC mixture (a well-known domain-forming mixture) [20]. A range of temperatures from 25 to 50°C was chosen to include the Tm of the pure lipids and the liquid immiscibility transition temperatures of the binary and ternary mixtures. We conducted fluorescence spectroscopy studies using two probes, DPH and Laurdan. DPH is used as a probe for the deep hydrophobic core of the acyl chain regions of the phospholipid bilayer, and Laurdan is used as a highly sensitive probe for the glycerol backbone of phospholipids. Measurements of time-resolved fluorescence anisotropy, intensity decay, DPH steady-state anisotropy and Laurdan spectral shifts were performed. To study the influence of the cholesterol content of the bilayer on the behavior of the segregated domains, Laurdan generalized polarization (Laurdan GP) microscopy measurements using two-photon excitation were performed in GUVs (giant unilamellar vesicles) of a 1∶1 DOPC/DPPC mixture [20].

Our results show that cholesterol incorporation into lipid vesicles induces changes in the structural and dynamic properties of the bilayer and that these changes are dependent on the depth and phase state of the bilayer. The Laurdan GP imaging results suggest that segregated liquid phases in GUVs model are stabilized by water molecules, which form bridges between cholesterol and PC heads.

## Results

To study the effect of cholesterol on the phospholipid lamella, three systems were used: pure DOPC, pure DPPC and a binary mixture of 1∶1 DOPC/DPPC (mol:mol%). Fluorescence spectroscopy measurements (Laurdan GP and lifetime, DPH anisotropy and lifetime) were taken using large unilamellar vesicles (LUVs) at 25, 37, 41 and 50°C. We also used Laurdan GP imaging to study the 1∶1 DOPC/DPPC mixture as a function of bilayer cholesterol content using a two-photon excitation microscope and giant unilamellar vesicles (GUVs).

### Spectroscopy Measurements

#### Steady-State fluorescence measurements

**Figure 1:**
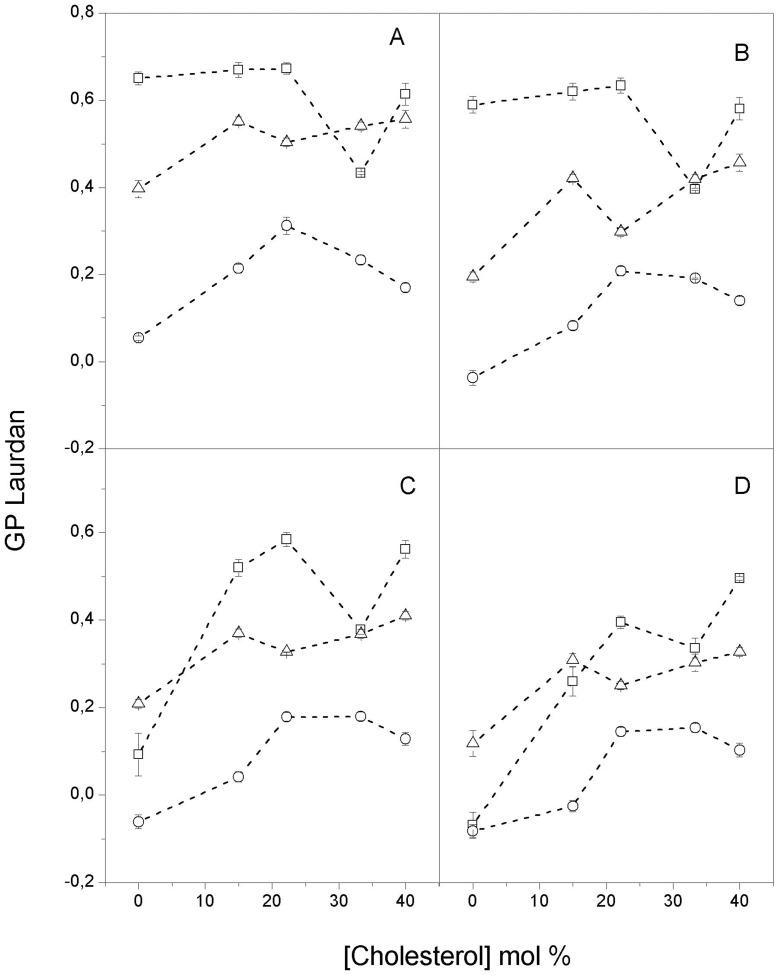
Laurdan generalized polarization: Laurdan generalized polarization in unilamellar vesicles of (-□-) DPPC; (-○-) DOPC and (-Δ-) DOPC/DPPC as a function of cholesterol content at (A) 20°C; (B) 37°C; (C) 45°C and (D) 50°C. Each point represents the average and standard error of triplicate data.

The effect of temperature on the three membrane systems and different cholesterol content was studied using DPH steady-state fluorescence anisotropy and Laurdan emission spectral shifts, which were evaluated from the generalized polarization measurements [21]. These fluorescent dyes locate at two different positions in the lipid bilayer; therefore, they can report the effect of cholesterol from these two locations: the deep hydrophobic core of acyl chain regions (DPH) and the hydrophobic-hydrophilic interface (Laurdan). The changes in DPH anisotropy with increasing cholesterol content in the bilayer follow the general trend previously published in the literature [22]: cholesterol decreases the order of lipids in the gel state (DPH anisotropy decrease) and increases the order of lipids in the liquid crystalline state. Laurdan emission spectral shifts (Fig. 1), which were inferred from GP changes, had an overall trend of blue shifts (GP increase) in the three systems, with the exception of the DPPC bilayer in the gel phase (25 and 37°C) where a slight decrease was observed (square in Fig. 1). All of the plots of the three types of vesicles showed non-monotonic behaviors at all of the temperatures.

#### Time-Resolved fluorescence measurements

**Figure 2:**
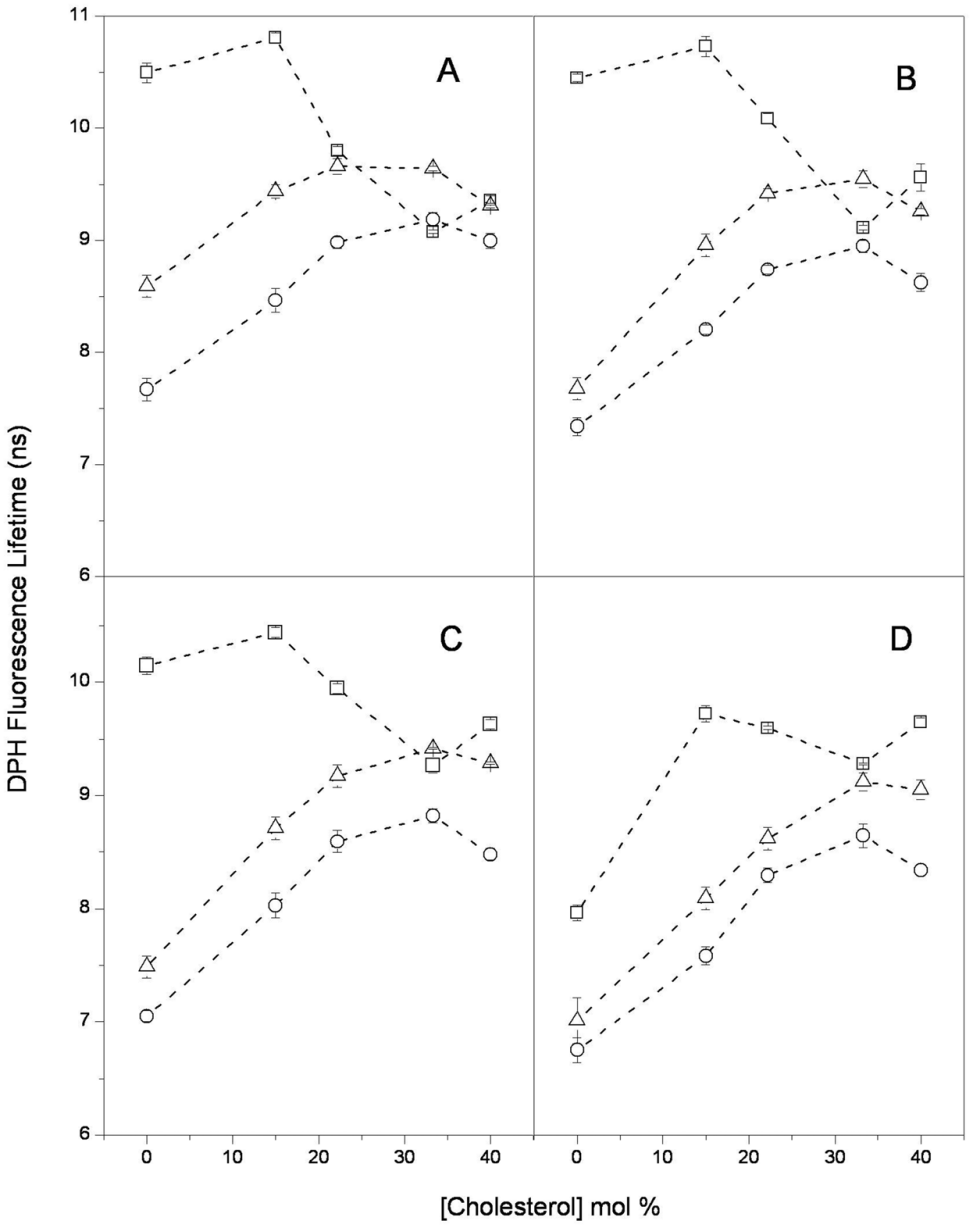
DPH fluorescence lifetime: DPH fluorescence lifetime distribution center in LUVs of (-□-) DPPC; (-○-) DOPC and (-Δ-) DOPC/DPPC as a function of cholesterol content at (A) 20°C; (B) 37°C; (C) 45°C and (D) 50°C. Each point represents the average and standard error of triplicate data.

**Figure 3:**
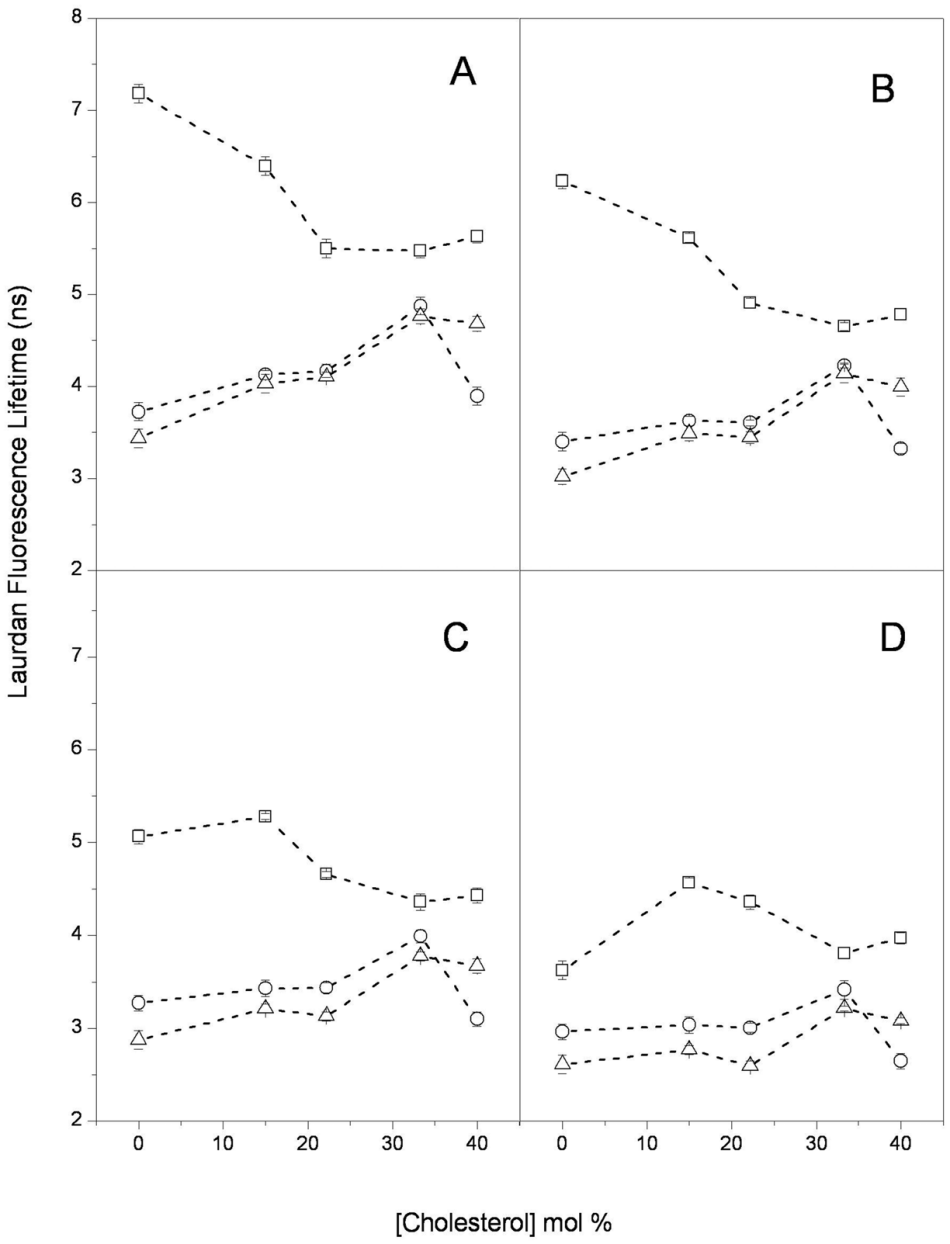
Laurdan fluorescence lifetime: Laurdan fluorescence lifetime distribution center in unilamellar vesicles of (-□-) DPPC; (-○-) DOPC and (-Δ-) DOPC/DPPC as a function of cholesterol content at (A) 20°C; (B) 37°C; (C) 45°C and (D) 50°C. Each point represents the average and standard error of triplicate data.

Fluorescence lifetime and anisotropy decay measurements of DPH and Laurdan embedded in the vesicle bilayers were also performed in the same systems (Figs. 2 and 3).

The lifetime data for DPH (Fig. 2) and Laurdan (Fig. 3) were analyzed using a Lorentzian distribution (methods); the center of the distribution τ_c_ is reported as the fluorophore lifetime. Fig. 2 shows the DPH fluorescence lifetimes for the three systems. In all three systems, the DPH lifetimes have non-monotonic changes as a function of cholesterol content. For DPPC (Tm = 41°C), an overall decrease in the lifetime occurred in the gel state (25 and 37°C, squares in Fig. 2A and B, respectively). At a slightly higher temperature, the Tm, this lifetime decrease is significantly lower. For 50°C (corresponding to liquid crystalline state), the lifetime increases with increasing cholesterol content (50°C, squares in Fig. 2D). In DOPC (Tm = −20°C) and the 1∶1 DOPC/DPPC mixture, the DPH lifetime increased consistently as the cholesterol content increased up to 33.3 mol% and decreased slightly above 40 mol% cholesterol.

The Laurdan fluorescence lifetimes as a function of cholesterol content are shown in Fig. 3A-D. For DPPC, two behaviors are observed depending on the temperatures. At 25 and 37°C (below Tm) (squares in Fig. 3A and 3B, respectively), as the cholesterol content increases, the Laurdan lifetime decreases and reaches a minimum value at 33.3 mol% cholesterol, above which a slight increase occurs. At the two higher temperatures, 41 (at Tm) and 50°C (above Tm), the lifetime initially increased up to 15 mol% cholesterol and then decreased to the mentioned minimum at 33.3 mol% cholesterol. For DOPC and the 1∶1 DOPC/DPPC mixture, the behavior is similar for all the temperatures: the Laurdan lifetime increases in proportion to the cholesterol content, reaches a maximum at 33.3 mol%, and then decreases, considerably in DOPC and slightly in the 1∶1 DOPC/DPPC mixture.

Comparing the general trends for the DPH (Fig. 2) and Laurdan (Fig. 3) lifetimes as a function of cholesterol content and temperature for the three samples provides some interesting observations:

- As expected from the Tm, for zero cholesterol content at all of the temperatures, the DPH and Laurdan lifetimes in DOPC (circles in Figures 2 and 3) are significantly lower than those in DPPC (squares in Figures 2 and 3). This difference decreases as the cholesterol content increases, which is particularly evident for DPH: the lifetime values become similar for all three systems at 33.3 mol% cholesterol and all of the temperatures.
- The DPH and Laurdan lifetimes in DOPC increase overall with cholesterol content at all of the temperatures (circles in Figs. 2 and 3). However, the behavior in DPPC (squares in Figs. 2 and 3) depends on the temperature: an overall increase in lifetime occurs if the lipid was initially in the liquid crystalline state (50°C), and a decrease in lifetime occurs if the lipid was initially in the gel state (25 and 37°C).
- In the 1∶1 DOPC/DPPC mixture, the DPH lifetime (triangles in Fig. 2) was intermediate to the values that were found for the individual components (DPPC and DOPC) at all of the temperatures. However, the Laurdan lifetime in the mixture (triangles in Fig. 3) was lower than the lifetimes in DPPC and DOPC, particularly with lower cholesterol content; the lowest value occurred at 40 mol% DOPC. Interestingly, the changes in both the DPH and Laurdan lifetimes with increasing cholesterol are similar in the mixture and in DOPC (circles and triangles, respectively, in Figs. 2 and 3).

The corresponding parameters from the analysis of the time-resolved anisotropy data are shown in Table 1 for DPH and Laurdan at 25 and 50°C. As seen in Table 1, *θ_1_* was lowest for DPH in the pure DPPC vesicles, whereas the highest values were found in the pure DOPC vesicles. The corresponding values for Laurdan in the DPPC vesicles were significantly higher than the values found for DPH. The opposite was found in the pure DOPC and the DOPC/DPPC vesicles (Table 1) with the exception of the DOPC/DPPC mixture at 50°C. The DPH fractional amplitude *f_1_*, which is related to *θ_1_* or R_1_, had a small value in the pure DPPC vesicles at low temperature and increased drastically with increasing temperature. Predictably, the highest values of the three systems were found in the DOPC vesicles at 25°C with a slight increase with temperature. Compared with DPH, the corresponding parameter for Laurdan had higher values in the DPPC vesicles and lower values in the DOPC vesicles at all of the temperatures, whereas in the mixture, higher values were found at low temperatures and the opposite was found at high temperatures. The order parameter from the DPH anisotropy decay had values within the expected ranges; the highest was in the pure DPPC vesicles in the gel state, with a drastic decrease in the liquid crystalline state. Low S values were found in the DOPC vesicles, and intermediate S values were found in the DOPC/DPPC vesicles. The corresponding values that were recovered from the Laurdan anisotropy decay in the pure DPPC vesicles are lower than the ones recovered from the DPH data; however, the opposite was found in the DOPC vesicles.

**Table 1:** Anisotropy decay parameters in unilamellar vesicles.

DPH												
[Chol]mol%	DPPC 25°C						DPPC 50°C					
	θ_1_(ns )	f_1_	R_1_(rad ns^−1^)	r_∞_	r_0_	S	θ_1_(ns )	f_1_	R_1_(rad ns^−1^)	r_∞_	r_0_	S
0	0.80±0.03	0.11	0.208±0.007	0.36	0.40	0.95	0.89±0.02	0.79	0.187±0.004	0.08	0.40	0.46
15	0.71±0.03	0.08	0.235±0.009	0.34	0.40	0.96	0.58±0.01	0.69	0.287±0.005	0.12	0.38	0.55
22.2	0.78±0.02	0.04	0.214±0.005	0.37	0.38	0.98	0.73±0.03	0.51	0.228±0.009	0.14	0.28	0.70
33.3	0.99±0.02	0.09	0.168±0.003	0.31	0.37	0.91	0.89±0.02	0.19	0.187±0.004	0.27	0.32	0.91
40	0.85±0.03	0.13	0.196±0.007	0.33	0.38	0.93	0.85±0.02	0.23	0.196±0.004	0.23	0.30	0.88
**[Chol]** **mol%**	**DOPC 25°C**						**DOPC 50°C**					
	**θ_1_** **(ns )**	**f_1_**	**R_1_** **(rad ns** ^−**1**^ **)**	**r_∞_**	**r_0_**	**S**	**θ_1_** **(ns )**	**f_1_**	**R_1_** **(rad ns** ^−**1**^ **)**	**r_∞_**	**r_0_**	**S**
0	3.45±0.05	0.94	0.048±0.001	0.02	0.32	0.25	1.48±0.02	0.97	0.113±0.001	0.01	0.30	0.18
15	3.74±0.06	0.84	0.045±0.001	0.05	0.31	0.40	1.39±0.08	0.88	0.120±0.007	0.04	0.33	0.35
22.2	2.65±0.07	0.65	0.063±0.001	0.11	0.34	0.60	1.14±0.07	0.79	0.146±0.009	0.06	0.29	0.46
33.3	3.00±0.07	0.45	0.056±0.001	0.17	0.31	0.74	1.61±0.08	0.60	0.104±0.007	0.12	0.30	0.63
40	2.51±0.08	0.44	0.066±0.002	0.18	0.32	0.75	1.27±0.05	0.61	0.131±0.005	0.12	0.31	0.62
**[Chol]** **mol%**	**DOPC/DPPC 25°C**						**DOPC/DPPC 50°C**					
	**θ_1_** **(ns )**	**f_1_**	**R_1_** **(rad ns** ^−**1**^ **)**	**r_∞_**	**r_0_**	**S**	**θ_1_** **(ns )**	**f_1_**	**R_1_** **(rad ns** ^−**1**^ **)**	**r_∞_**	**r_0_**	**S**
0	2.98±0.08	0.62	0.056±0.002	0.13	0.34	0.62	1.07±0.05	0.90	0.156±0.007	0.03	0.31	0.31
15	2.10±0.05	0.46	0.079±0.002	0.20	0.37	0.74	0.93±0.03	0.79	0.179±0.006	0.08	0.39	0.45
22.2	2.50±0.09	0.41	0.067±0.002	0.22	0.37	0.77	0.99±0.04	0.78	0.168±0.007	0.09	0.40	0.47
33.3	2.16±0.05	0.29	0.077±0.002	0.24	0.34	0.71	0.82±0.04	0.61	0.203±0.009	0.15	0.39	0.62
40	2.40±0.05	0.26	0.069±0.001	0.25	0.34	0.86	1.28±0.05	0.37	0.130±0.005	0.19	0.30	0.80
**Laurdan**												
**[Chol]** **mol%**	**DPPC 25°C**						**DPPC 50°C**					
	**θ_1_** **(ns )**	**f_1_**	**R_1_** **(rad ns^−1^)**	**r_∞_**	**r_0_**	**S**	**θ_1_** **(ns )**	**f_1_**	**R_1_** **(rad ns^−1^)**	**r_∞_**	**r_0_**	**S**
0	1.02±0.06	0.27	0.163±0.009	0.27	0.37	0.85	1.27±0.05	0.89	0.131±0.005	0.04	0.33	0.35
15	2.59±0.01	0.28	0.064±0.001	0.26	0.35	0.85	1.77±0.03	0.59	0.094±0.001	0.14	0.33	0.65
22.2	2.70±0.01	0.32	0.062±0.001	0.23	0.33	0.82	2.02±0.06	0.59	0.083±0.002	0.13	0.32	0.64
33.3	2.32±0.04	0.41	0.072±0.001	0.20	0.34	0.77	1.66±0.01	0.68	0.100±0.001	0.10	0.34	0.54
40	2.38±0.02	0.38	0.070±0.001	0.21	0.34	0.79	1.71±0.02	0.67	0.097±0.001	0.11	0.35	0.57
**[Chol]** **mol%**	**DOPC 25°C**						**DOPC 50°C**					
	**θ_1_** **(ns )**	**f_1_**	**R_1_** **(rad ns** ^−**1**^ **)**	**r_∞_**	**r_0_**	**S**	**θ_1_** **(ns )**	**f_1_**	**R_1_** **(rad ns** ^−**1**^ **)**	**r_∞_**	**r_0_**	**S**
0	2.05±0.02	0.78	0.081±0.001	0.07	0.33	0.46	1.15±0.03	0.89	0.145±0.004	0.03	0.31	0.33
15	2.04±0.01	0.85	0.082±0.001	0.05	0.34	0.38	1.09±0.05	0.94	0.152±0.007	0.02	0.31	0.24
22.2	2.20±0.04	0.78	0.076±0.001	0.07	0.32	0.47	1.11±0.05	0.93	0.150±0.007	0.02	0.31	0.26
33.3	2.27±0.02	0.68	0.073±0.001	0.11	0.34	0.56	1.21±.0.02	0.91	0.138±0.002	0.03	0.33	0.30
40	2.41±0.02	0.76	0.069±0.001	0.07	0.34	0.45	1.25±0.02	0.87	0.133±0.002	0.04	0.31	0.36
**[Chol]** **mol%**	**DOPC/DPPC 25°C**						**DOPC/DPPC 50°C**					
	**θ_1_** **(ns )**	**f_1_**	**R_1_** **(rad ns** ^−**1**^ **)**	**r_∞_**	**r_0_**	**S**	**θ_1_** **(ns )**	**f_1_**	**R_1_** **(rad ns** ^−**1**^ **)**	**r_∞_**	**r_0_**	**S**
0	2.23±0.05	0.76	0.075±0.002	0.09	0.36	0.50	1.23±0.01	0.83	0.136±0.001	0.06	0.33	0.43
15	2.91±0.03	0.77	0.057±0.001	0.09	0.36	0.50	1.60±0.03	0.90	0.104±0.002	0.03	0.34	0.30
22.2	2.73±0.02	0.72	0.061±0.001	0.10	0.37	0.52	1.60±0.02	0.91	0.104±0.002	0.03	0.34	0.30
33.3	2.37±0.02	0.61	0.070±0.001	0.14	0.35	0.63	1.34±0.02	0.84	0.124±0.002	0.05	0.34	0.38
40	2.92±0.03	0.61	0.057±0.001	0.14	0.36	0.62	1.40±0.01	0.81	0.119±0.001	0.07	0.35	0.45

Effect of modification of membrane cholesterol content. The phase and modulation data are analyzed with a hindered rotation decay model*^a^.*

a *r*_0_ refers to the time 0 anisotropy. *r_∞_* is the residual anisotropy at infinite time. *f*_1_ is the fraction of the observed anisotropy decay associated with the fast depolarization process. *Θ_1_* is the fast rotational correlation time. *R_1_* refers to the fast rotational rate. *S* is the order parameter. The reduced chi-squared (*χ* ^2^) value ranges from 0.3 up to maximum values of 8.0. The *r*_0_ and *r_∞_* errors were all <0.03; the *f*_1_ and *S* errors were all <0.06. The errors represent the correlated 67% confidence limits of the reduced *χ* ^2^.

At all temperatures, the incorporation of cholesterol into vesicles results in a non-monotonic behavior of the rotational correlation times of both probes. The corresponding parameter for DPH showed an overall decrease with cholesterol incorporation in DOPC-containing vesicles and no variation in DPPC vesicles, whereas the Laurdan parameter had an overall increase in all of the vesicles. The incorporation of cholesterol induced an overall decrease in the Laurdan rotational rate and an increase in the DPH rate except for in the DPPC vesicles, where the overall change is negligible (Table 1). As the cholesterol content increased, the DPH fractional amplitude *f_1_* had a drastic monotonous decrease in all of the vesicles (except for the DPPC vesicles at 25°C, where *f_1_* remained almost unchanged). The corresponding parameter for Laurdan in all of the vesicles had much smaller changes and remained nearly unchanged in the DOPC vesicles. A drastic increase in the order parameter “S”, which was recovered from the DPH anisotropy decay, was observed upon incorporation of cholesterol into all of the vesicles (except for the DPPC vesicles in the gel state, where the value remained unchanged). The Laurdan corresponding order parameter had slight changes upon cholesterol incorporation only in the DPPC vesicles (decreases in the gel state and increases in the liquid crystalline state) and in DOPC/DPPC vesicles at 25°C (a slight increase).

### Two-photon Microscopy Measurements

Previous microscopy studies have reported that the ternary mixture of DOPC/DPPC:cholesterol has micrometer-sized segregated domains [23]–[25]. Under some conditions, the Laurdan GP images of these GUVs showed areas with different GP, indicating the presence of segregated domains. To study the behavior of coexisting lipid domains with different concentrations of cholesterol, we performed Laurdan GP microscopy images measurements in this ternary system.

GP images of Laurdan-labeled GUVs that were composed of DOPC/DPPC (1∶1 mol:mol in all cases) with cholesterol contents of 0, 15, 22.2, 33.3 and 40 mol% were obtained at different temperatures between 20 and 60°C.

**Figure 4:**
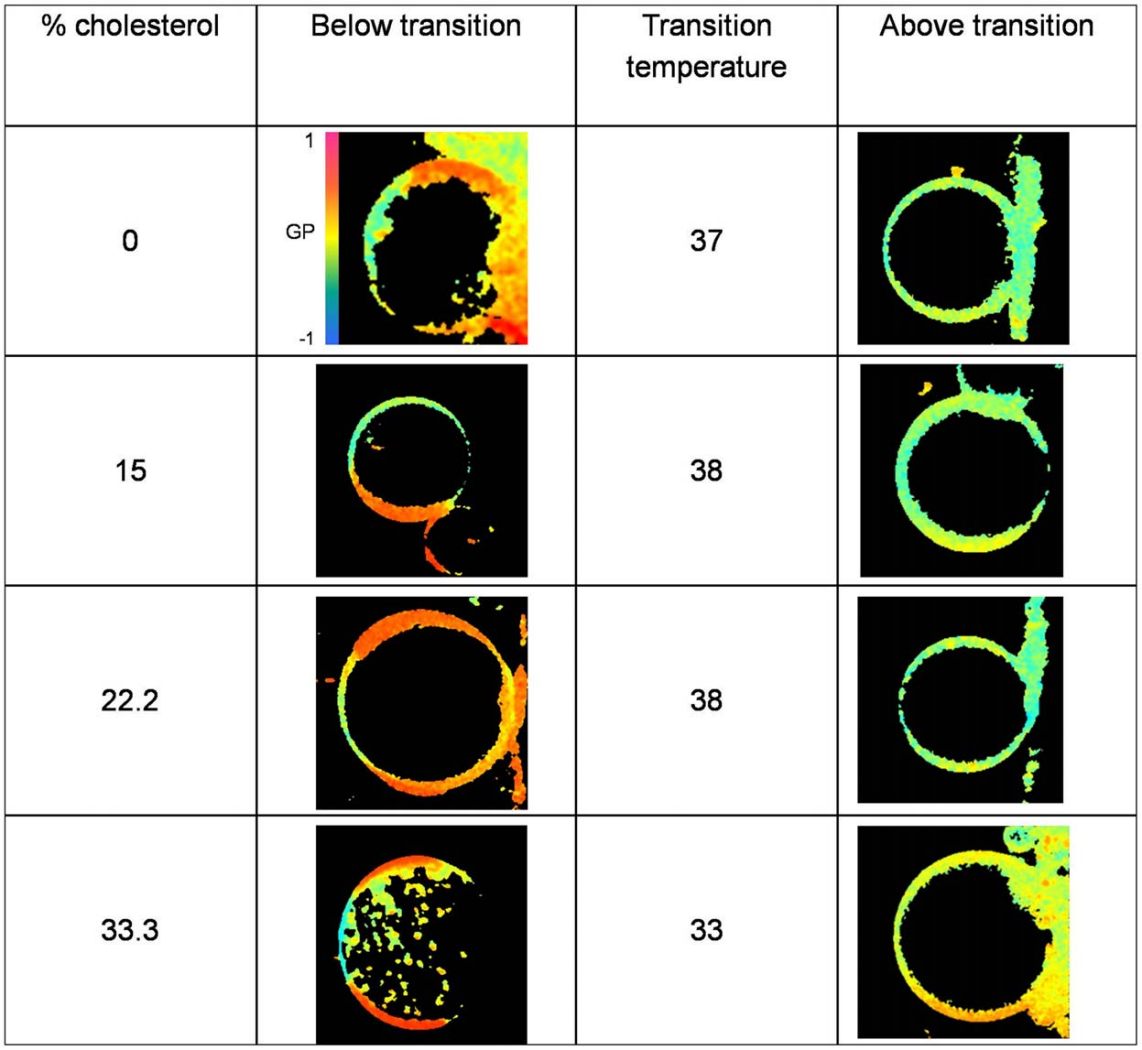
Laurdan GP images: Laurdan generalized polarization images of the DOPC/DPPC (1∶1 mol%) mixtures with different cholesterol content. The immiscibility transition temperature is indicated for each mixture. Different GP values are represented by an artificial color bar.

**Figure 5:**
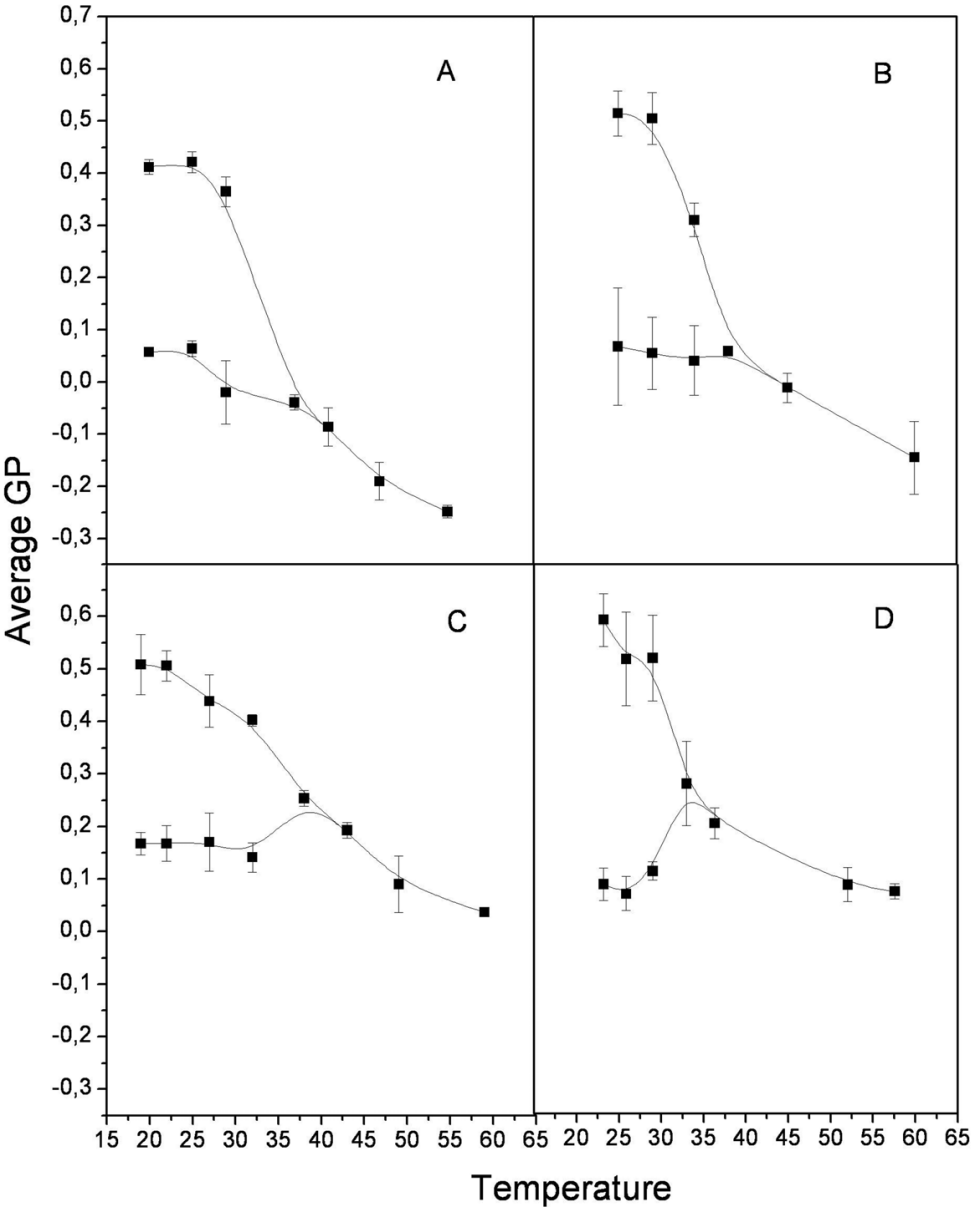
Average Laurdan GP values: Thermotropic behavior of average Laurdan GP histograms values in the DOPC/DPPC (1∶1 mol%) mixture with (A) 0, (B) 15 mol%, (C) 22.2 mol% and (D) 33.3 mol% cholesterol.

**Figure 6:**
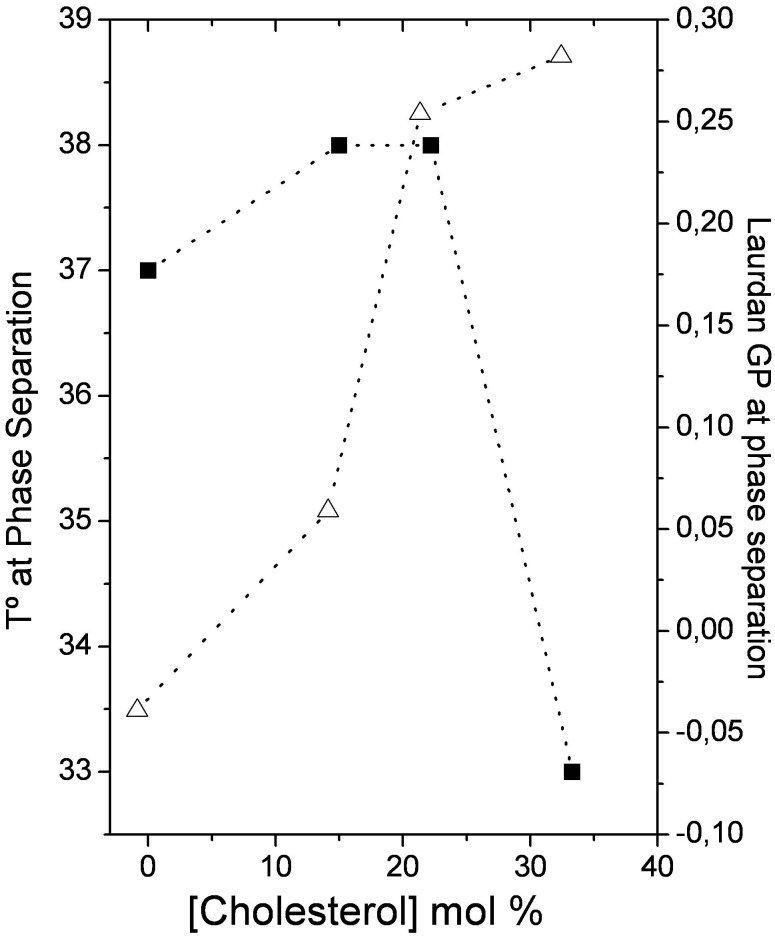
Temperature at phase separation: Immiscibility transition temperatures (-▪-) and Laurdan GP value (-Δ-) at which the immiscibility occurs as a function of bilayer cholesterol content.

**Figure 7:**
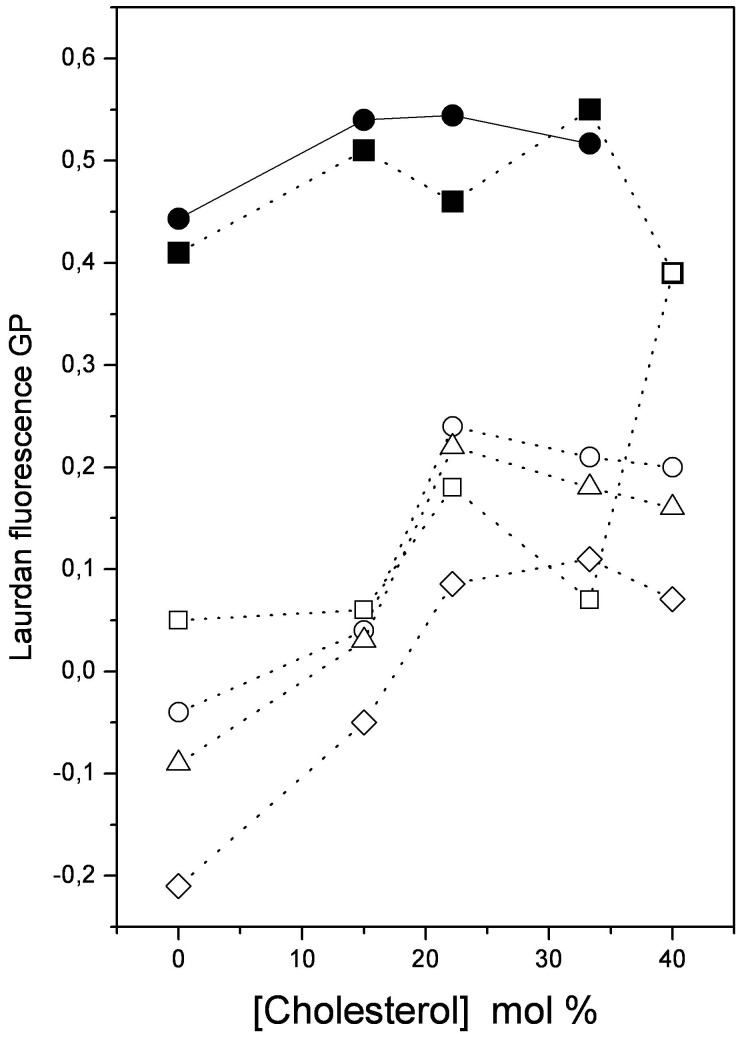
Laurdan GP values in GUVs: Laurdan fluorescence GP in GUVs of DOPC/DPPC (1∶1 mol%) as a function of cholesterol content, obtained from two-photon microscopy GP images at different temperatures. The symbols correspond to (-▪-)the high GP value at 25°C, (-□-) the low GP value at 25°C, (-○-) the GP value at 37°C, (-Δ-) the GP value at 41°C, (-◊-) the GP value at 50°C and (-•-) the GP value in LUVs of DOPC/DPPC (1∶1 mol%) as a function of cholesterol content.

Figure 4 shows Laurdan GP images of 1∶1 DOPC/DPPC GUVs with 0, 15, 22.2 and 33.3 mol% cholesterol below and above the domain coexistence temperatures. The transition temperature was estimated from the curves in Fig. 5: one GP value was observed at temperatures where there was no phase separation, and two GP values (high and low GP) were observed at temperatures in the immiscibility range. The mixtures with 40 mol % cholesterol showed no distinct areas in the measured temperature range, which is in contrast to previous reports [20].This difference may be explained by the sample preparation: we obtained the GUVs by starting with LUVs in buffer, rather than in chloroform solutions as previously reported [20]. Fig. 6 summarizes the data from the Laurdan GP microscopy; the GP values at the phase separation and at the temperature where the transition occurred are shown as a function of bilayer cholesterol content. Note that at 25°C, two GP values are plotted for each cholesterol concentration. The immiscibility transition temperatures of the mixtures and the Laurdan GP value at which the immiscibility occurs are plotted as a function of cholesterol concentration in Fig. 7. While the former showed a biphasic pattern with a maximum at approximately 22.2 mol % cholesterol, the latter increased for the entire concentration interval.

## Discussion

We studied the effects of cholesterol content on the structural and dynamic properties of bilayers that were composed of a 1∶1 DOPC/DPPC mixture and of the individual components; large unilamellar vesicles (LUVs) were used as model membranes. We used DPH and Laurdan to obtain information at different depths in the lamella as a function of cholesterol content. Giant unilamellar vesicles, GUVs, of the mixtures were also examined using two-photon excitation microscopy and Laurdan generalized polarization measurements.

The fluorescence lifetimes of DPH and Laurdan provide information about water penetration into the bilayer [26], [27]. A comparison of the 1∶1 DOPC/DPPC mixture with the pure DOPC system indicates that the formation of the mixture decreases bilayer hydration at the deep hydrophobic level of the bilayer (from the DPH lifetime) and slightly enhances water penetration at the level of the glycerol backbone (from the Laurdan lifetime).

The general trend is increased DPH and Laurdan lifetimes as a function of the cholesterol content in the DOPC and 1∶1 DOPC/DPPC vesicles (Figs. 2 and 3); this increase is interpreted as the decrease in the bilayer hydration at all levels that is due to cholesterol [28], [29]. The same effect is observed in the DPPC LUVs in the liquid-crystalline state (50°C); however, an overall increase in hydration is observed in the gel state. We also found that the information from lifetime changes concurs with that from the Laurdan spectral shifts (Fig. 1).

The time-resolved anisotropy data for DPH indicate that cholesterol incorporation induces an increase in the fluorophore rotational rate; the increased rate indicates that there is more order at the deep hydrophobic core of the acyl chain region in the bilayers in all of the samples (except for DPPC). This concomitant ordering and rotational rate increase appears contradictory at first glance. However, Beensdorff and co-workers [30] reported a similar result for the studies of hydrostatic pressure in lipid vesicles. The authors proposed that the pressure-induced constraints that are imposed upon the rotational wobbling amplitude of the fluorophore may lead to an increase in the rate of motion within the restricted cone; this increased rate of motion occurs if the wobble angle in which the probe can rotate on a nanosecond time scale decreases upon pressurization. Similarly, Pasenkiewicz-Gierula and co-workers proposed a decrease in the available cone angle as a result of cholesterol incorporation into DPPC and DMPC bilayers [31]. The unexpectedly large value for the fast rotational rate found in DPPC compared with the other two vesicles that did not have cholesterol (Table 1) may be explained similarly, that is, a higher rate of fluorophore motion within a smaller wobble angle. In contrast, the Laurdan results indicate that cholesterol incorporation induces a decrease in the rotational rate of the probe in all of the samples. The considerable decrease of the Laurdan rotational rate in the DPPC vesicles with cholesterol incorporation is interesting.

A comparison of the DPH and Laurdan data suggests that the changes induced by the incorporation of cholesterol into the bilayer are different for the deep hydrophobic phospholipids acyl chains domain than for the shallow region at the hydrophilic-hydrophobic interface. Because the rotational rate of these probes reflects the orientational dynamics of the surrounding phospholipids, we propose that cholesterol incorporation induces an increase in orientational dynamics at the deep region of the phospholipid acyl chains and a corresponding orientational dynamics decrease at the region close to the lipid polar head groups. Our conclusion is in agreement with Bartucci and co-workers [32], who proposed that the methyl terminal of the chain moves more freely in the presence of cholesterol. Cholesterol incorporation affects the structure: the order increases at the deep acyl chain level (except in DPPC LUVs in the gel state where no change is observed), whereas the order decreases at the glycerol backbone level (although other effects were also observed). The lifetime data from Laurdan and DPH show that the overall hydration changes produced by the addition of cholesterol are similar at both bilayer levels in the DOPC and DOPC/DPPC vesicles. These changes can be explained in the context of the results reported by Marsh [29]. He suggested that chain insaturation increases water permeation at the level of the headgroups of the acyl chains, reducing the effect of cholesterol in this region. In the DPPC vesicles, the effect at both depths depends on the state of the lipid phase: an overall increase in the fluid state and an overall decrease in the gel state (Figs. 2 and 3).

Previous results from microscopy studies [20], [23]–[25] show the segregation of macro domains in GUVs of DOPC/DPPC/cholesterol, and the phase diagram for the mixture is well established. We used this system and Laurdan GP microscopy to examine the behavior of these domains as a function of cholesterol content.

As the temperature decreased in the Laurdan GP experiments, the GUVs changed from one homogeneous phase (one GP value) to two co-existing phases (two GP values, one from each domain) (Fig. 5). The immiscibility transition temperature of the mixtures initially increased slightly with bilayer cholesterol content (Fig. 6). However, a significant decrease occurred at concentrations higher than 22.2 mol% of cholesterol, which is in agreement with previous results [20]. For the same range of cholesterol concentrations, the GP value at which the immiscibility occurs increases with a similar slope until 22.2 mol% (Fig. 6). The sudden decrease in the transition temperature at 22.2% cholesterol indicates that the stability of a bilayer with segregated liquid phases decreases as cholesterol content increases. A possible explanation for this behavior is that cholesterol may decrease the overall hydration of the bilayer [28], [29]. Thus, it has been proposed that in cholesterol-containing lipid bilayers, H-bonds are formed between the OH- group from Chol (OH-Chol) and the oxygen atoms from PC (particularly the γ-chain carbonyl oxygen atom) and between OH-Chol and water [16], [17]. However, molecular simulations by Pasenkiewicz-Gierula and co-workers [19] predict that the predominant interaction between Chol and PC are via water bridges and charge pairs more than via direct hydrogen bonding. We suggest that decreasing water content in the bilayer could destabilize the hydrogen bond network, influencing the stability of the Lo phase, which shows lower hydration.

The Laurdan GP of the DOPC/DPPC mixture in GUVs and LUVs as a function of cholesterol content had a similar overall increase with non-monotonic changes for all of the temperatures. Thus, we expected that GP values obtained from LUVs (measured in a cuvette, at 25°C) would be between the ones obtained for the individual segregated domains at this temperature. However, the values are closer to the ones obtained for the ordered phase. To rationalize this unexpectedly high value, we hypothesize that LUVs have slower dynamics at the shallow depth due to a more constrained structure even though LUVs have more hydration due to their more pronounced curvature. In addition, the difference in diameter between the two types of vesicles, from tens of micrometers in GUVs to hundreds of nanometers in LUVs, implies a considerable difference in bilayer curvature, thus influencing lipid interactions, ordering, dynamics, etc.

Our results show non-monotonic changes in fluorescence parameters with increasing amounts of cholesterol and indicate that the changes induced by cholesterol are different at the deep hydrophobic level than at the shallow region of the hydrophilic-hydrophobic interface. In particular, the orientational dynamics increase at the deep hydrophobic level of the lamella and decrease at the shallow depth, which is close to the polar lipid headgroups. For overall structural changes, there is an ordering at the deep level and a disordering at the shallow level. Another contribution of our work is based on a proposal that bilayer Chol content increases that are higher than 22.2 mol% induce changes in lipid organization due to the concomitant decrease in the water content; because water participates in the hydrogen bond network that bridges Chol-lipid headgroups, increasing cholesterol content weakens the stability of this network and hence of the segregated liquid phases.

Most of the measured parameters exhibit a non-monotonic dependence on cholesterol concentration. Thus, a sterol superlattice model has been proposed by Chong [33]. Although this model may not be universally accepted, it explains and predicts this type of dependence accurately. The study of sterol superlattice formation was not an aim of our work. To study superlattice formation, the systems must be examined using small increments of cholesterol mole fraction.

## Materials and Methods

### Materials

The nonionic surfactant n-dodecyl-β-D-maltoside (DOM) was obtained from Anatrace, Inc. (Maumee, OH, USA). 1,6-Diphenyl-1,3,5-hexatriene (DPH) and 2-(dimethylamino)-6-lauroylnaphthalene (Laurdan) were obtained from Molecular Probes (Eugene, OR, USA). Bio-Beads SM-2 were obtained from Bio-Rad (Irvine, CA, USA). All of the lipids and cholesterol were obtained from Avanti Polar Lipids (Alabaster, AL, USA).

### Large Unilamellar Vesicle (LUV) Preparation

The lipids were suspended in DOM and incubated with hydrophobic polystyrene beads, Bio-Beads SM2 (0.2 g/mL), to remove the detergent. To overcome lipid adsorption by the beads, the Bio-Beads were pre-saturated by incubating them with an excess of a small unilamellar vesicle (SUVs) suspension of the same lipid or lipid mixture [34]. The LUV samples were prepared at least 15 days prior to the measurements and were stored sealed with nitrogen, which was replenished periodically. DPH and Laurdan were incorporated into 0.4 mM total lipid LUV suspensions and adjusted to a final concentration of 0.5 µM with stock solutions of DMSO and ethanol, respectively, and incubated at 37°C for 45 min. The amount of cholesterol and phospholipids was determined using cholesterol (Cholesterol E) and phospholipid (Phospholipid C) reagents, respectively, that were obtained from Wako Chemicals (Richmond, VA).

### Giant Unilamellar Vesicle (GUV) preparation

The GUVs were grown using the conventional electroforming method [35], [36] but using stock solutions of LUVs of different lipid mixtures in buffer. The vesicles were formed by first spreading 2 µL of the LUV stock solution onto the platinum wires of the growing chamber. The stock solutions (approximately 0.2 mg/mL total lipid) contained LUVs of mixtures of 1∶1 DOPC/DPPC with increasing amounts of cholesterol. The sample was allowed to rest for 15 minutes at room temperature in the dark. A microscope cover slip was glued on the bottom of the growing chamber and thermostated with a circulating water bath at the growing temperature (normally 10°C over the corresponding transition temperature of the lipid). When the desired temperature was reached, buffer (1 mM Tris, pH 7.4) of the same temperature was added, and the platinum wires were connected to a function generator set to 1 V amplitude at 10 Hz frequency. After the vesicle was formed, the AC field was turned off and followed by a cooling process to each of the measuring temperatures. A CCD video camera (CCD-Iris, Sony, Tokyo, Japan) was used to follow the growing vesicles. The temperature of the thermostated sample chamber was monitored using a temperature probe (model 400B, Omega, Stamford, CT) that was placed in the chamber near the platinum wire. We used a method previously described in detail [37], [38]. Before the measurements, Laurdan was added from a stock solution in DMSO to give a concentration of 0.25 µM, and the solution was incubated for at least 10 minutes.

### Fluorescence Spectroscopy Measurements

Steady-state and time-resolved fluorescence measurements were performed on a K-2 multifrequency phase and modulation spectrofluorometer (ISS, Champaign, IL, USA). The instrument was equipped with a Glan-Thompson polarizer. For both probes, the excitation light was from a modulatable ISS 375 nm LED laser. The emission was measured through Schott KV-399 and WG-420 long band-pass filters. The lifetime measurements were conducted with the polarizer oriented in the “magic angle” condition [39]. To measure anisotropy decay, differential phase angles and modulation ratios were obtained from the parallel and perpendicular orientations of the sinusoidal polarized emission. The phase and modulation values were obtained as previously described [40], [41]. Dimethyl-POPOP (1,4-bis [2]4-methyl-5-phenyloxazoly benzene) in ethanol (τ = 1.45 ns) was used as a reference for the intensity decay. The generalized polarization of Laurdan, which was utilized to assess its fluorescence spectral shifts, was measured as previously described [21]. The data shown represent the mean values and standard error of measurements of two independent samples.

### Data Analysis

The time-resolved fluorescence data were analyzed using the Globals Unlimited software package (Laboratory for Fluorescence Dynamics, University of Illinois at Urbana-Champaign, Urbana, IL). The fitting function for the lifetime measurements was the sum of a continuously distributed Lorentzian component and a discrete component, which was fixed at 0.01 ns to account for scattered light [41]. The anisotropy decay data were fitted to a hindered rotator model of anisotropy decay (see equation 1), which is based on the “wobble-in-cone” model [42]; the model included a hindered rotation component, i.e., two rotational correlation times, where the second rotational correlation time, θ_2_, was fixed at a large value (1 ms) relative to the lifetime.
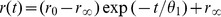
(1)


In equation (1), *r*_0_ is the amplitude of the anisotropy decay at time 0, *θ_1_* is the fast rotational correlation time of the anisotropy decay and *r_∞_* is the residual anisotropy at infinite time. *θ_1_* is related to the fractional amplitude *f_1_*, i.e., the fraction of molecules associated with *θ_1_*, which is f_1_ = 1 - *r*_∞_/*r*_0_. The fluorophore rotational rate R_1_ is related to *θ_1_* by R_1_ = 1/6*θ_1_* and S, the mean second rank-order parameter of the fluorescent probe in the bilayer, is calculated from the residual and initial anisotropy values using S = (r_∞/_r_0_)^1/2^ [43].

### Laurdan GP Images

The images were collected on a scanning two-photon fluorescence microscope at the Laboratory for Fluorescence Dynamics (University of California, Irvine) as described previously [44], [45]. A mode-locked titanium-sapphire laser (Mira 900, Coherent, Palo Alto, CA) pumped by a frequency-doubled Nd:Vanadate laser (Verdi, Coherent) and set to 780 nm was used as the two-photon excitation light source. Two intensity images were collected simultaneously using a two-channel detection system with a beamsplitter and interference filters (Ealing 490 and Ealing 440). These images were then recombined to form the images of Laurdan generalized polarization, GP, using the SimFCS software (Laboratory for Fluorescence Dynamics, University of California, Irvine, CA). The histograms were smoothed and normalized to the maximum signal by nonlinear regression using a Gaussian function. The GP value shown is the histogram average for each Gaussian.

## References

[1] McIntosh TJ, Simon SA (2006). Role of bilayer material properties in function and distribution of membrane proteins.. Annu Rev Biophys Biomol Struct.

[2] Yeagle P (1989). Lipid regulation of cell membrane structure and function.. ASEB J.

[3] Barenholz Y (2001). Cholesterol and other membrane active sterols: from membrane evolution to “rafts”. Prog Lipid Res.

[4] Levi M, Baird BM, Wilson PV (1990). Cholesterol modulates rat renal brush border membrane phosphate transport.. J Clin Invest.

[5] Ho C, Stubbs CD (1992). Hydration at the membrane protein-lipid interface.. Biophys J.

[6] Yeagle PL (1985). Cholesterol and the cell membrane.. Biochim Biophys Acta.

[7] Erilov DA, Bartucci R, Guzzi R, Marsh D, Dzuba SA (2004). Librational Motion of Spin-Labeled Lipids in High-Cholesterol Containing Membranes from Echo-Detected EPR Spectra.. Biophys J.

[8] Isaev NP, Syryamina VN, Dzuba SA (2010). Influence of Cholesterol on Molecular Motions in Spin-Labeled Lipid Bilayers Observed by Stimulated ESE.. Appl Magn Reson.

[9] Ipsen JH, Karlstrom G, Mouritsen OG, Wennerstrom H, Zuckermann J (1987). Phase equilibria in the phosphatidylcholine-cholesterol system.. Biochim Biophys Acta.

[10] Nielsen M, Miao L, Ipsen JH, Zuckermann M, Mouritsen OG (1999). Off-lattice model for the phase behavior of lipid-cholesterol bilayers.. Phys Rev E.

[11] Ohvo-Rekilä H, Ramstedt B, Leppimäki P, Slotte JP (2001). Cholesterol interactions with phospholipids in membranes.. Prog Lip Res.

[12] Simons K, Ikonen E (1997). Functional rafts in cell membranes.. Nature.

[13] Martinez-Seara H, Róg T, Karttunen M, Vattulainen I, Reigada R (2010). Cholesterol Induces Specific Spatial and Orientational Order in Cholesterol/Phospholipid Membranes.. PLoS ONE.

[14] McElhane RN (1982). The use of differential scanning calorimetry and differential thermal analysis in studies of model and biological membranes.. Chem Phys Lip.

[15] Hao M, Mukherjee S, Maxfield FR (2001). Cholesterol depletion induces large scale domain segregation in living cell membranes.. Proc Natl Acad Sci USA.

[16] Robinson AJ, Richards WG, Thomas PJ, Hann MM (1995). Behavior of cholesterol and its effect on head group and chain conformations in lipid bilayers: a molecular dynamics study.. Biophys J.

[17] Tu K, Klein ML, Tobias DJ (1998). Constant-pressure molecular dynamics investigation of cholesterol effects in a dipalmitoylphosphatidylcholine bilayer.. Biophys J.

[18] Bhattacharya S, Haldar S (2000). Interactions between cholesterol and lipids in bilayer membranes. Role of lipid headgroup and hydrocarbon chain–backbone linkage.. Biochim Biophys Acta.

[19] Pasenkiewicz-Gierula M, Róg T, Kitamura K, Kusumi A (2000). Cholesterol Effects on the Phosphatidylcholine Bilayer Polar Region: A Molecular Simulation Study.. Biophys J.

[20] Veatch SL, Keller SL (2003). Separation of Liquid Phases in Giant Vesicles of Ternary Mixtures of Phospholipids and Cholesterol.. Biophys J.

[21] Parasassi T, De Stasio G, d’Ubaldo A, Gratton E (1990). Phase fluctuation in phospholipid membranes revealed by Laurdan fluorescente.. Biophys J.

[22] Lentz BR, Barrow DA, Hoechli M (1980). Cholesterol-phosphatidyl choline interactions in multilamellar vesicles.. Biochemistry.

[23] Sanchez S, Tricerri M, Gratton E (2007). Interaction of high density lipoprotein particles with membranes containing cholesterol.. J Lip Res.

[24] Bernardino de la Serna J, Perez-Gil J, Simonsen AC, Bagatolli LA (2004). Cholesterol rules: direct observation of the coexistence of two fluid phases in native pulmonary surfactant membranes at physiological temperatures.. J Biol Chem.

[25] de Almeida RF, Borst JW, Fedorov A, Prieto M, Visser AJ (2007). Complexity of Lipid Domains and Rafts in Giant Unilamellar Vesicles Revealed by Combining Imaging and Microscopic and Macroscopic Time-Resolved Fluorescence.. Biophys J.

[26] Zannoni C, Argioni A, Cavatorta P (1983). Fluorescence depolarization in liquid crystals and membrane bilayers.. Chem Phys Lipids.

[27] Bernsdorff C, Winter R, Hazlett TL, Gratton E (1995). Influence of cholesterol and β-sitosterol on the dynamics behavior of DPPC as detected by TMA-DPH and PyrPC fluorescence. A fluorescence lifetime distribution and time-resolved anisotropy study.. Ber Bunsenges Phys Chem.

[28] Parasassi T, Di Stefano M, Loiero M, Ravagnan G, Gratton E (1994). Cholesterol modifies water concentration and dynamics in phospholipid bilayers: a fluorescence study using laurdan probe.. Biophys J.

[29] Marsh D (2002). Membrane water penetration profiles from spin labels.. Eur Biophys J.

[30] Bernsdortf C, Wolf A, Winter R, Gratton E (1997). Effect of hydrostatic pressure on water penetration and rotational dynamics in phospholipid-cholesterol bilayers.. Biophys J.

[31] Pasenkiewicz-Gierula M, Subczynski WK, Kusumi A (1990). Rotational diffusion of a steroid molecule in phosphatidylcholine-cholesterol membranes: fluid-phase microimmiscibility in unsaturated phosphatidylcholine-cholesterol membranes.. Biochemistry.

[32] Bartucci R, Erilov DA, Guzzi R, Sportelli L, Dzubac SA (2006). Time-resolved electron spin resonance studies of spin-labelled lipids in membranes.. Chem Phys Lipids.

[33] Chong PLG, Zhu W, Venegas B (2009). On the lateral structure of model membranes containing cholesterol. Biochim. Biophys.. Acta.

[34] Rigaud JL, Mosser G, Lacapere JJ, Olofsson A, Levy D (1997). Bio-beads: an efficient strategy for two-dimensional crystallization of membrane proteins.. J Struct Biol.

[35] Angelova MI, Dimitrov DS (1986). Liposome electroformation. Faraday Discuss Chem.. Soc.

[36] Angelova MI, Soléau S, Méléard Ph, Faucon F, Bothorel P (1992). Preparation of giant vesicles by external AC electric fields. Kinetics and applications.. Prog Colloids Polym Sci.

[37] Sanchez SA, Bagatolli LA, Gratton E, Hazlett TL (2002). A two-photon view of an enzyme at work: Crotalus atrox venom PLA2 interaction with single-lipid and mixed-lipid giant unilamellar vesicles.. Biophys J.

[38] Sanchez SA, Tricerri MA, Gunther G, Gratton E, Mendez-Villas A, Diaz J (2007). Laurdan generalized polarization: from cuvette to microscope..

[39] Spencer RD, Weber G (1969). Measurement of subnanosecond fluorescence lifetimes with a cross-correlation phase fluorometer.. Ann NY Acad Sci.

[40] Gratton E, Jameson D, Hall RD (1984). Multifrequency phase and modulation fluorometry.. Ann Rev Biophys Bioeng.

[41] Alcala JR, Gratton E, Prendergast FG (1987). Resolvability of fluorescence lifetime distributions using phase fluorometry.. Biophys J.

[42] Kinosita K, Kawato S, Ikegami A (1977). A theory of fluorescence polarization decay in membranes.. Biophys J.

[43] Lakowicz JR (2006). Principles of Fluorescence Spectroscopy. New York: Springer.. 954 p.

[44] Yu W, So PT, French T, Gratton E (1996). Fluorescence generalized polarization of cell membranes: a two-photon scanning microscopy approach.. Biophys J.

[45] Bagatolli LA, Gratton E (2000). Two Photon Fluorescence Microscopy of Coexisting Lipid Domains in Giant Unilamellar Vesicles of Binary Phospholipid Mixtures.. Biophys J.

